# Erythroderma: A Retrospective Study of 212 Patients Hospitalized in a Tertiary Center in Lower Silesia, Poland

**DOI:** 10.3390/jcm13030645

**Published:** 2024-01-23

**Authors:** Katarzyna Kliniec, Aleksandra Snopkowska, Magdalena Łyko, Alina Jankowska-Konsur

**Affiliations:** 1 Department of Dermatology, Venereology and Allergology, Wroclaw Medical University, 50-368 Wroclaw, Poland; katarzyna.kliniec@gmail.com (K.K.); alina.jankowska-konsur@umw.edu.pl (A.J.-K.); 2 Student Research Group of Experimental Dermatology, Department of Dermatology, Venereology and Allergology, Wroclaw Medical University, 50-368 Wroclaw, Poland; aleksandra.snopkowska@student.umw.edu.pl

**Keywords:** erythroderma, etiology, clinical data, laboratory findings, treatment

## Abstract

Erythroderma is a condition characterized by erythema affecting at least 90% of the skin surface area. It can be caused by various underlying conditions. Due to nonspecific clinical and laboratory findings, determining the cause may pose a challenge. In the retrospective study, we identified 212 patients hospitalized for erythroderma in the Department of Dermatology, Venereology, and Allergology at Wroclaw Medical University between January 2012 and March 2022. Clinical, laboratory, and histopathological features, as well as the management of patients, were studied. The median age of adults was 61 years (IQR = 47–68). The most common causes of erythroderma were psoriasis (*n* = 49, 24.01%), followed by atopic dermatitis (AD) (*n* = 27, 13.23%), and cutaneous T-cell lymphomas (CTCL) (*n* = 27, 13.23%). Despite laboratory tests and histopathological examination, the etiology of erythroderma remained undetermined in 39 cases (19.12%). In 70.59% of patients, it was the first episode of erythroderma, while 29.41% experienced a recurrent episode. Regardless of the etiology of erythroderma, patients were most frequently treated with systemic antihistamines (146 cases, 71.57%) and systemic steroids (132 cases, 64.71%). Patients with idiopathic erythroderma constitute the greatest diagnostic and therapeutic challenge, requiring particularly thorough evaluation.

## 1. Introduction

Erythroderma is an inflammatory condition characterized by erythema affecting at least 90% of the skin surface area. It may be a clinical manifestation of various diseases, including inflammatory and immunobullous dermatoses, drug reactions, hematologic and solid organ malignancies, infections and infestations, or connective tissue disorders [1,2,3].

Erythroderma is a potentially life-threatening disease that requires hospitalization and intensive treatment, constituting a huge burden for patients and their families, as well as for medical staff and the health system [4].

The presented study aimed to analyze clinical, laboratory, and histopathological features as well as the management of patients diagnosed and treated for erythroderma hospitalized in the dermatology department of a tertiary referral hospital.

## 2. Materials and Methods

Two hundred twelve records of patients with erythroderma admitted to the Department of Dermatology, Venereology, and Allergology at Wroclaw Medical University between January 2012 and March 2022 were reviewed. We selected dermatoses that, according to the literature, can proceed with erythroderma, e.g., psoriasis vulgaris, atopic dermatitis, cutaneous T-cell lymphomas (CTCL), and drug-induced erythroderma. Using the International Classification of Diseases, 10th revision (ICD-10) codes B86, C84.0, C84.1, L10.2, L20.8, L20.9, L21, L23, L27, L30.9, L40.0, L40.1, L40.2, L40.4, L40.5, L40.8, L40.9, L43, L44.0, L51.1, L51.2, L51.8, L51.9, L56, and Q80, the hospital electronic database was searched to identify the records. The analyzed data included information on gender and age, duration of hospitalization, previous hospitalizations, systemic diseases, drug intake, past erythrodermic episodes, the cause of the current episode of erythroderma, and clinical data during the episode (occurrence of lymphadenopathy). Treatment methods and laboratory tests (blood count, inflammatory markers (erythrocyte sedimentation rate (ESR), C reactive protein (CRP), procalcitonin), immunoglobulin E (IgE), IgE panel, albumin, β2-microglobulin) have also been studied.

### Statistical Analyses

Statistical analyses were performed using STATISTICA v. 13 software (StatSoft Inc., Tulsa, OK, USA). The quantitative variables were described using median, range, interquartile range (IQR), and means ± standard deviations (SD). The relationships between the groups and the factors were examined using the Mann–Whitney U test. The Kruskal–Wallis one-way analysis of variance (ANOVA) was used to compare more than two groups. For categorical variables, the χ^2^ test was performed. Values of *p* < 0.05 were considered statistically significant.

## 3. Results

### 3.1. Demographic Data

Of the 212 patients studied, the vast majority (204) were adults with a median age of 61 years (IQR = 47–68). Among them, 135 (66.18%) were male and 69 (33.82%) were female. The youngest patient was 2 years old, and the oldest was 96 years old. The pediatric population consisted of 8 patients with a median age of 6 years. As the number of pediatric population was insufficient for statistical analysis, cases of erythroderma in children were excluded from statistical analysis and discussed separately.

The median length of hospitalization was 10 days (IQR = 8–15). The median length of hospitalization did not differ according to erythroderma cause.

### 3.2. The Cause of Erythroderma

The cause of erythroderma was known in 167 patients (81.86%). Among them, psoriasis was the most common (*n* = 49, 24.01%), followed by atopic dermatitis (*n* = 27, 13.23%) and cutaneous T-cell lymphomas (CTCL) (*n* = 27, 13.23%). The most common reason for drug-induced erythroderma was allopurinol (*n* = 5, 25.8%) followed by anticonvulsants (*n*= 4, 16.7%). The most common cause of erythroderma in the pediatric population was atopic dermatitis (*n* = 5, 62.5%). In patients with idiopathic erythroderma, we performed a follow-up. In nine patients, we were able to provide a diagnosis at first evaluation, and in our analysis, patients were included in appropriate groups. Among 48 patients, five were diagnosed with CTCL, two with eczema, and two with drug-induced erythroderma. Over the 10 years individual cases of erythroderma in the course of pityriasis rubra pilaris (PRP) (*n* = 8/204; 3.92%), generalized pustular psoriasis (GPP) (*n* = 4/204; 1.96%), congenital ichthyosis (*n* = 2/204; 0.98%), toxic epidermal necrolysis/Stevens-Johnson syndrome (TEN/SJS) (*n* = 2/204; 0.98%), seborrheic dermatitis (*n* = 1/204; 0.49%), crusted scabies (*n* = 1/204; 0.49%), pemphigus foliaceus (PF) (*n* = 1/204; 0.49%) were observed. Therefore, we grouped the data as other causes and did not draw any conclusions according to this group. In 70.59% of patients, it was the first episode of erythroderma, while 29.41% experienced a recurrent episode. In patients diagnosed with CTCL (*n* = 15, 55.56%) and eczema (*n* = 9, 45.0%), erythroderma most often occurred in the past. Characteristics of patients according to etiology are presented in Table 1. Characteristics of patients with erythroderma according to rare etiologies are presented in Table S1.

### 3.3. Age

In the analyzed group including the most frequent underlying dermatoses responsible for erythroderma, the highest median age was observed for eczema (Me = 68, IQR = 63–73), followed by drug reaction (Me = 67, IQR = 58–72), CTCL (Me = 66, IQR = 59–77), idiopathic(Me = 64, IQR = 59–70), plaque psoriasis (Me = 51, IQR = 41–61) and atopic dermatitis (Me = 40, IQR = 21–54). There was a statistically significant difference in median age between the groups (*p* < 0.01). Post hoc tests revealed that the median age of patients with AD and psoriasis was significantly lower than the median age of patients with CTCL, eczema, drug-induced erythroderma, and idiopathic erythroderma (*p* < 0.01 for all groups).

### 3.4. Clinical Data during the Episode of Erythroderma

Histopathological examination was performed in 96 (47.06%) patients. In 59 (61.46%) patients, the diagnosis was made based on histopathology. However, in 37 (38.54%) cases, histopathological examination was inconclusive, and a diagnosis was not established.

Lymphadenopathy was noted in 42 (20.59%) patients. Among them, enlarged lymph nodes were observed in 21 (50%) CTCL patients, 11 (26.2%) patients with idiopathic erythroderma, four (9.5%) AD patients, and six single cases (14.29%) in the remaining groups. In 79 (38.73%) cases, there was no data on whether lymph nodes were enlarged or not. There was a statistically significant difference in the occurrence of lymphadenopathy between groups (*p* < 0.001). Lymphadenopathy occurred significantly more often in individuals with erythroderma in the course of CTCL. Among the 27 patients with erythrodermic CTCL included in the study, 21 (77.78%) presented with lymphadenopathy.

### 3.5. Laboratory Findings

Among the laboratory parameters, a significant association was observed on admission between the cause of erythroderma and the level of ESR (*p* = 0.0039), CRP (*p* = 0.0000), and immunoglobulins IgE (*p* = 0.0008). A significantly higher level of ESR was observed in psoriasis than in AD and drug-induced erythroderma (*p* < 0.001). Moreover, CRP was significantly lower in CTCL than in psoriasis and drug-induced groups. Total IgE was significantly higher in AD than in psoriasis, eczema, drug-induced, and idiopathic erythroderma (*p* < 0.001 for all groups). Detailed data are shown in Table 1.

### 3.6. Comorbidities

The prevalent comorbidities among all patients were hypertension (*n* = 83, 40.69%), heart disease (*n* = 43, 21.08%), anemia (*n* = 37, 18.14%), and diabetes (*n* = 35, 17.16%). Similarly, subjects with idiopathic erythroderma were most often accompanied by hypertension (*n* = 19, 51.35%), followed by anemia (*n* = 12, 32.43%), and diabetes (*n* = 10, 27.03%). Psoriatic arthritis was additionally diagnosed in 4 patients with psoriasis vulgaris. Single cases of obesity; past infections, neurological, gastrointestinal, rheumatic, kidney, lung, and thyroid diseases were also observed. There were no significant differences in the prevalence of the aforementioned comorbidities between the groups. However, hypertension was observed less frequently in erythroderma in the course of AD (*n* = 5; 18.52%).

### 3.7. Pre-Hospitalisation Treatment

Before admission, eight patients (16.33%) with erythrodermic psoriasis underwent steroid treatment. Among them, five (10.20%) were treated with topical steroids, and one received a combination of both topical and systemic steroids. For the remaining cases, information on whether it was topical or systemic therapy was unavailable. Methotrexate was administered to six patients (12.24%), acitretin to eight (16.33%), and two (4.08%) patients who received cyclosporine. Phototherapy was administered in eleven cases (22.45%), dithranol in three (6.12%), and biological treatments in two (4.08%). A subset of therapies was combined. Data on pre-hospitalization treatment in twenty-one patients (42.86%) was incomplete, and specific treatment details were not collected for this group. Additionally, eleven patients (22.45%) with erythrodermic psoriasis received treatment for comorbidities before hospitalization, predominantly with antihypertensive drugs. This included five patients (10.20%) receiving angiotensin-converting enzyme inhibitors, two receiving calcium channel blockers (4.08%), five receiving beta-blockers (10.20%), and five receiving diuretics (10.20%). Moreover, five patients were treated with anticoagulants (10.20%) and one with antidiabetic medication (2.04%). However, due to the lack of data in many cases and the small sample sizes in these groups, further statistical analyses were not performed, as it could be considered insignificant.

In erythroderma due to atopic dermatitis, nine (33.33%) patients received steroid treatment, including two (7.41%) with topical steroids. For other cases, information on whether it was systemic or topical steroids was lacking. Four (14.81%) patients underwent phototherapy, six (22.22%) were treated with immunosuppressants (including two with cyclosporine and one with acitretin), and in three (11.11%) patients, the specific immunosuppressive drug administered could not be established. Additionally, nine (33.33%) patients received antihistamines. The treatments were combined. Data was incomplete or unspecific in twelve (44.44%) cases. Among the eleven (40.74%) patients with erythroderma caused by atopic dermatitis, treatment for comorbidities was administered, with antihypertensive drugs predominating, followed by beta-2 mimetics. Specifically, three (11.11%) patients were treated with angiotensin-converting enzyme inhibitors, four (14.81%) with beta-blockers, and two (7.41%) with diuretics. Beta-2 mimetics were administered to four (14.81%) patients. Additionally, two (7.41%) patients received anticoagulants, two (7.41%) were prescribed antidiabetic drugs, and two (7.41%) were given antidepressants.

Patients with CTCL were treated with steroids in nineteen cases (70.37%), with four (14.81%) of them receiving systemic steroids and one receiving (3.70%) both topical and systemic treatment. Data concerning the type of steroid used for fifteen (55.55%) patients was incomplete. Immunosuppressants were administered to thirteen (48.14%) patients, including four (14.81%) treated with methotrexate and one (3.70%) with cyclosporine. For the remaining cases, data on the specific immunosuppressive drug used were incomplete. Other cases included three receiving retinoids (11.11%) and fifteen (55.55%) undergoing phototherapy. Some therapies were combined. Additionally, nine (33.33%) were treated with antihistamines. In the case of CTCL, six (22.22%) patients underwent therapy for comorbidities, primarily for hypertension, with one (3.70%) patient receiving angiotensin-converting enzyme inhibitors and two (7.41%) receiving beta-blockers. Furthermore, one (3.70%) patient received anticoagulants, another was prescribed antidiabetic drugs, and one underwent antidepressant therapy. In five (18.52%) cases, data were incomplete, and authors could not establish pre-hospitalization treatment.

In the eczema patient group, immunosuppressants were administered in five (23.81%) cases, including one (4.76%) treated with methotrexate, one (4.76%) with ciclosporin, and one (4.76%) with azathioprine. Steroids were administered in ten (47.61%) cases, with data in five (23.81%) cases being nonspecific, in three (14.29%) involving systemic treatment, and in two (9.52%) topical steroids. Phototherapy was used in six (28.57%) cases, and some therapies were combined. Moreover, antihistamines were used in six (28.57%) patients. Data for nine (42.86%) cases were incomplete. Eleven (52.38%) patients received therapy for comorbidities, primarily for hypertension. This included angiotensin-converting enzyme inhibitors administered in three (14.29%) cases, beta-blockers prescribed to three (14.29%) patients, and calcium channel blockers and diuretics administered to two (9.52%) patients each. Additionally, one (4.76%) patient received anticoagulants, and two (9.52%) were prescribed antidiabetic drugs.

In other preexisting dermatoses, as well as in psoriasis, a substantial amount of data was lacking. Small sample sizes were collected in groups concerning specific pre-hospitalization treatment, and further statistical analysis was not undertaken. The authors did not delve further into this area in this publication.

### 3.8. Management of Erythroderma

Regardless of the cause, in the group of patients with erythroderma, systemic antihistamines (146 cases, 71.57%) were the most frequently selected therapeutic approach, followed by systemic steroids (132 cases, 64.71%). The subsequent options included antibiotics (77 cases, 37.75%), methotrexate (60 cases, 29.41%), cyclosporine A (37 cases, 18.14%), phototherapy (35 cases, 17.15%), acitretin (16 cases, 7.84%) and azathioprine (2 cases, 0.98%).

The results highlighted the significant differences in the administration of systemic steroids between the groups (*p* < 0.001). In patients with psoriatic erythroderma, systemic steroids were used less frequently than in patients with other erythroderma (*p* < 0.001).

Systemic antihistamines were predominantly used in AD (96.27%), drug-induced (86.36%), eczema (80.95%), and idiopathic (79.49%) erythroderma. A statistically significant difference was observed in the use of systemic antihistamines between groups (*p* < 0.001). Systemic antihistamines were more frequently used in the aforementioned groups than in CTCL and psoriasis.

Regarding phototherapy, no significant differences were observed among the examined groups (*p* > 0.05).

MTX was most frequently used among CTCL (59.26%) and psoriasis (42.86%) patients. Statistically significant differences were found in the usage of MTX medication between the psoriasis group and the groups of patients with idiopathic erythroderma, AD, eczema, and drug-induced skin reactions (*p* < 0.01). Moreover, statistically significant differences were found between CTCL and AD and drug-induced skin reaction groups (*p* < 0.01). However, no statistically significant differences in the usage of MTX were observed among the remaining groups.

Cyclosporine A was the most common treatment option for patients with AD (*n* = 14; 51.85%). There were statistically significant differences between groups (*p* < 0.001). This therapy was more frequently used in the AD group compared with the pairs of all other erythroderma causes.

There was also a significant difference in systemic antibiotic use between groups (*p* = 0.006). This therapeutic option was frequently used in psoriasis, AD, and drug-induced erythroderma, while in CTCL and eczema-caused erythroderma, antibiotics were administered sparingly. Patients with elevated CRP and neutrophil levels were significantly more likely to receive antibiotics.

**Figure 1:**
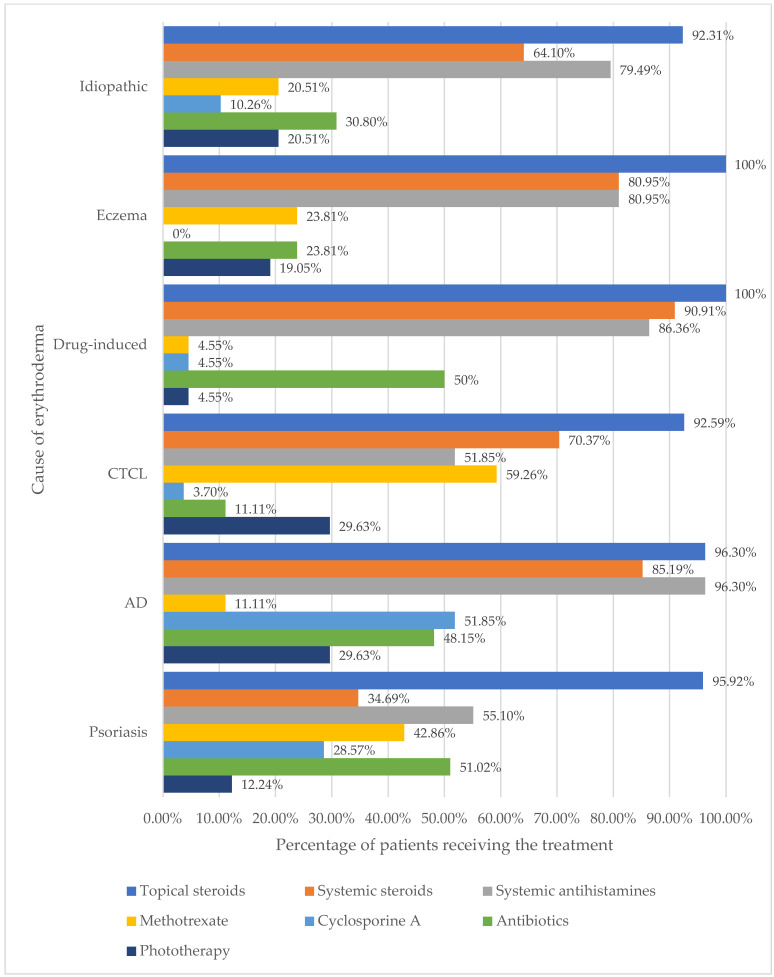
The treatment is according to the cause of erythroderma. CTCL—primary cutaneous T-cell lymphoma, AD—atopic dermatitis.

Detailed data on the management of erythroderma in each group is shown in Figure 1.

Patients in our study did not receive biological treatment during hospitalization. However, several patients were treated with biologics before hospital admission or were subsequently enrolled in a biologic treatment program. One pediatric patient in our sample received biological treatment (dupilumab) for atopic dermatitis before hospitalization. Two patients with psoriasis vulgaris were treated with biologics. One received ustekinumab, and in the other case adalimumab was initially administered, but due to an unsatisfactory response, the drug was changed to ustekinumab with positive results. One patient with psoriatic arthritis was treated with secukinumab.

The authors conducted a database search of patients enrolled in the biologic treatment program to determine whether patients included in the study were subsequently recruited into the program. Four patients from our study were identified as part of the biologic therapy program: three with psoriasis vulgaris and one with atopic dermatitis.

One patient with psoriasis began treatment with ustekinumab in 2015 with a BSA of 91 and a PASI of 35.4. The last follow-up in 2023 provided information on improvement to a BSA of 0.1 and a PASI of 2.5. In one case, adalimumab treatment in 2017 was given to a patient with a BSA of 35 and a PASI of 18.9, but due to lack of the expected response, the decision was made to switch medications. The patient received risankizumab with a BSA response of 0.5 and a PASI of 1.6. Another patient from our study was included, but the records only contain information about the first visit during which adalimumab was administered, and there was no follow-up visit, making it impossible to determine the response.

One pediatric patient with atopic dermatitis received dupilumab in 2022. Treatment was initiated with a BSA of 100 and an EASI of 68.4. At the 2023 follow-up visit, the EASI was 0.

To control most cases of erythroderma, a systemic therapy regimen was used, which consists of a total of 4 mg of clemastine intravenously daily in two divided doses and intravenous hydrocortisone at an initial dose of 200 mg daily and then, depending on the patient’s condition, reducing or maintaining the dose. Usually, 200 mg of hydrocortisone was administered for 2 to 3 days, then the dose was reduced to 100 mg for the next 2 to 3 days, and the treatment was finished with intramuscular methylprednisolone in a single dose of 80 mg in depot form as an adjunct to hydrocortisone therapy. The second most frequently used antihistamine after clemastine was hydroxyzine at a dose of 25 mg per day. Among the antibiotics, the most frequently used was amoxicillin with clavulanic acid in a daily dose of 2 g. Among immunosuppressive drugs, the dose range of methotrexate was from 7.5 mg to a maximum of 30 mg per week, cyclosporine A was used in the dose range of 200 to 400 mg per day, and azathioprine in the dose of 150 mg per day.

## 4. Discussion

Patients with erythroderma constitute a heterogeneous group, which in most cases is a challenge both in terms of diagnosis and treatment. For this reason, we wanted to analyze patients’ outcomes during the hospitalization due to erythroderma.

In our study, we observed cases of erythroderma in both adults and children. The median age of the adult population in our study is in close agreement with the results of other studies, as is the median age determined using the etiology of erythroderma, particularly for psoriatic erythroderma, drug-induced and CTCL-induced erythroderma, and idiopathic erythroderma [1,2,3,4,5,6]. The age of patients with erythroderma secondary to CTCL, eczema, or idiopathic is higher. The youngest group was patients with AD. Male patients outnumbered females (1.96:1), which also agrees with reports from other authors where the male-to-female ratio varies between 1.5:1 and 2.2:1 [3,7,8]. In their report, Mathew and Sreedevan observed that erythroderma occurs almost four times more often in men than females (male-to-female ratio—3.6:1); however, the authors did not address the possible reason for such a pronounced male predominance [6].

We found that almost half of the patients (46.57%) developed erythroderma due to a preexisting skin condition. In other studies, this data varied between 38% and 74.6% [3,5,9,10]. That is why collecting a patient’s medical history plays such an important role.

During the study period, the median length of hospitalization for all patients from the Dermatology Department was 6 days, while for patients with erythroderma, it was much longer—10 days. The longer the hospitalization duration is, the higher financial costs for the hospital are, which makes erythroderma even more demanding both for health workers and the health system in general.

### 4.1. Psoriasis

Psoriasis vulgaris was the most common preexisting skin disease in our study population (24.02%, *n* = 49). Similar results (32.7%) were obtained by Mathew and Sreedevan [6]. Among the patients diagnosed with psoriatic erythroderma in our study, 91.84% were previously diagnosed with psoriasis vulgaris, while only 8.16% received a new diagnosis based on histological examination. In the world literature, about 1–2% of psoriasis patients experience at least one episode of erythroderma throughout their lives [11,12]. In the Miyashiro and Sanches study involving 309 patients, nearly 80% of them had a positive history of psoriasis vulgaris [2]. In another report, César et al. undertook a retrospective analysis of 103 patients with erythroderma, which also identified plaque psoriasis as the most common cause of this symptom [5]. Other researchers have made similar observations [1,3,6,8].

### 4.2. Atopic Dermatitis

The second most common cause of erythroderma was atopic dermatitis (AD) (13.24%). Among all 27 patients with AD erythroderma, 24 of them had a previous history of AD, and three other patients were newly diagnosed. Previous studies found AD as one of the most frequent reasons for erythroderma as well [2,6]. Higher immunoglobulin E levels were observed in AD 19,100 UI/mL compared to other causes of erythroderma, such as psoriasis vulgaris, drug reaction, and eczema. Miyashiro and Sanches made similar observations—the median IgE in the group of patients with AD-induced erythroderma was 24,600 UI/mL and was higher than the median IgE in other patients [2]. Askin et al. found elevated IgE in drug-induced and AD-related erythroderma [8]. Ohga et al. observed increased IgE values in AD patients as well, but also in cases of chronic idiopathic erythroderma [13].

### 4.3. CTCL

Of all 27 patients diagnosed with CTCL erythroderma, 14 of them had a previous known history of mycosis fungoides (MF) or Sezary’s syndrome (SS). In the case of 13 other patients, histological examination was decisive to give an accurate diagnosis. The patients with newly diagnosed CTCL were previously diagnosed with adult-onset atopic dermatitis (*n* = 2), psoriasis vulgaris (*n* = 1), and systemic lupus erythematosus (*n* = 1). In a study by Miyashiro and Sanches, SS was responsible for erythroderma in 12.3% of subjects, while MF was associated with erythroderma in 5.5% of cases [2]. Similar findings were provided by César et al., who reported that SS caused erythroderma in 12.3% of patients and MF was responsible for 5.5% of cases [5]. Additionally, Mathew and Sreedevan reported 12 cases of erythroderma due to MF, representing 3.2% of the study population [6]. It should be emphasized that the incidence of CTCL is significantly lower than the incidence of psoriasis vulgaris or AD; thus, the number of patients with this rare condition and erythroderma is lower. However, it is the most frequent malignant neoplasm responsible for erythroderma [4]. The median age in our study population was 66 years. In comparison, Miyashiro and Sanches reported a median age of 58 years for erythroderma caused by MF and 62 years for SS [2]. Rakowska et al. reported a mean age of 58 years for CTCL-induced erythroderma, while César et al. found a mean age of 69.4 years for erythroderma caused by malignancies (MF, SS, B-cell chronic lymphocytic leukemia) [5,14].

### 4.4. Drug Reaction

Erythroderma caused by drug reaction was observed in 10.78% of patients. Of all 22 patients diagnosed with drug-induced erythroderma, 5 of them had a previous history of allergic reactions. The observed incidence of a drug reaction as a cause of erythroderma is in accordance with the literature [2,5,6,8]. In a study conducted by Miyashiro and Sanches, it was found that 12.3% of patients experienced drug-induced erythroderma [2]. César et al. reported a comparable incidence, with 18.4% of affected subjects, which is similar to the findings of Askin et al. (17%) [5,8]. In addition, Mathew and Sreedevan observed drug-induced erythroderma in 6.5% of cases [6]. Drug-induced erythroderma may occur days or weeks after exposure, and it evolves quickly [1,15,16]. The number of drugs can be the causative factor [1,2]. The most common cause of drug-induced erythroderma in our sample was allopurinol (*n* = 5; 20.8%) and anticonvulsants (*n* = 4; 16.7%), such as carbamazepine (*n* = 3; 12.5%) and lamotrigine (*n* = 1; 4.2%). Table S2 presents the characteristics and timing of the development of drug-induced erythroderma. This agrees with the findings of other authors [3,5]. The above-mentioned medications are some of the most common agents responsible for severe drug-induced skin reactions, such as drug reaction with eosinophilia and systemic symptoms (DRESS) and Stevens-Johnson syndrome/toxic epidermal necrolysis (SJS/TNT) [17]. In a study by Tee et al., allopurinol was the most common medication causing severe cutaneous adverse reactions (SCAR), including SJS, TNT, SJS/TNT overlap, and DRESS [18]. These drug-induced skin reactions can lead to erythroderma [19]. Isolated cases of drug-induced erythroderma, according to our observations, were caused by venlafaxine (*n* = 1; 4.2%), tolperison (*n* = 1; 4.2%), telaprevir (*n* = 1; 4.2%), terbinafine (*n* = 1; 4.2%), bromhexine (*n* = 1; 4.2%), fenofibrate (*n* = 1; 4.2%). In previously published literature, single studies report telaprevir and terbinafine as possible causes of erythroderma [20,21,22]. In the remaining patients (*n* = 7; 29.2%), we were unable to determine which drug was the direct trigger. It seems that it may be primarily attributed to the multimorbidity of the subjects and the diverse array of medications they were concurrently taking. This group included patients who were simultaneously treated for various conditions, such as cardiovascular, neurological, psychiatric, and metabolic diseases. Identifying a specific causative factor may have been challenging due to the complexity of these interactions. Predominantly, polypharmacy was associated with the number and severity of comorbidities and older age of patients. What is more, drugs commonly used in comorbidities could also induce erythroderma, according to some studies [23,24,25,26,27,28]. Cases of β-blocker-induced erythroderma (e.g., bisoprolol, metoprolol) have also been reported in the literature [29,30]. Moreover, erythroderma can (rarely) be caused by metformin, insulin, and other diabetes drugs [25,26,27,28].

### 4.5. Eczema

Eczema was the cause of erythroderma in 21 (10.29%) patients. This is one of the most common causes of this symptom reported in the literature [2,5,6,8]. The median age in this group was significantly higher compared to other causes. No statistically significant differences were observed in laboratory results between the eczema and other groups.

### 4.6. Idiopathic Erythroderma

In 19.12% of patients, it was impossible to establish a clear cause of erythroderma. This percentage is close to results from other studies [2]. In the literature, idiopathic cases vary between 6.1% and up to 38% [2,6,10,31,32,33,34,35,36]. Ohga et al., in the study of erythrodermic patients from Fukuoka University Hospital Dermatology Department, described 23 cases of chronic idiopathic erythroderma (CIE) [13]. The group of patients with CIE was primarily elderly men with a mean age of 74.7 years old. In the study by Miyashiro and Sanches, the median age of patients with idiopathic erythroderma was 68 years, and they were mainly men [2]. Our results agree with this observation, as the majority of undiagnosed patients in our sample were men, and their median age was 63.5 years. The researchers emphasized that cases of idiopathic erythroderma cause may be as yet undiagnosed cases of CTCL and that patients with CIE are at increased risk of developing CTCL such as MF or SS [4,7,31,33,37,38]. In the study of 38 patients with idiopathic erythroderma, four of them evolved to MF during the follow-up period, and nine were suspected of having this condition [37]. Pal and Haroon have also observed the evolution of erythroderma with unknown etiology in CTCL [35]. Other authors stated that the high number of cases of CTCL erythroderma in their patients was the result of advanced diagnostic tools (tertiary referral center for CTCL) in patients who may have formerly been identified as idiopathic erythroderma [2].

### 4.7. Remaining Causes of Erythroderma

In addition to the above-mentioned causes of erythroderma, our patients were diagnosed with erythroderma triggered by the following diseases: pityriasis rubra pilaris (PRP) (8/204; 3.92%), generalized pustular psoriasis (GPP) (4/204; 1.96%), congenital ichthyosis (2/204; 0.98%), TEN/SJS (2/204; 0.98%), seborrheic dermatitis (1/204; 0.49%), crusted scabies (1/204; 0.49%), pemphigus foliaceus (PF) (1/204; 0.49%). According to available knowledge, all of these dermatoses detected in our patients are also described by worldwide researchers as possible, rare erythroderma triggers [1,39,40].

### 4.8. Lymphadenopathy

Lymph node enlargement was found in 20.59% of the study group, of which 83.3% were men, and the median age was 60 years. It occurred significantly more often in individuals with CTCL erythroderma and affected 77.78% of them. This was followed by patients with idiopathic erythroderma (28.20%). It is worth noting that in 38.73% of the cases, there was no data on whether lymph nodes were enlarged or not. Reports of lymphadenopathy in patients with erythroderma vary in the literature, with percentages ranging from 12.5% to 55.5% [5,6,8,9,31,32,33,34,35]. In a study on idiopathic erythroderma by Sigurdsson et al., 68% of patients had lymphadenopathy [38]. Miyashiro and Sanches noticed lymph node enlargement in 53.5% of patients [2]. They described that the highest percentage of lymphadenopathy occurred in patients with erythroderma due to AD (69.2%), followed by MF (66.7%), idiopathic (64%), and SS (63.2%) [2]. Researchers could obtain such a high number of patients with lymph node enlargement because, in addition to the clinical examination, they also used biopsy and CT scans to check the presence of lymphadenopathy.

### 4.9. Laboratory Findings

In our study, we found some differences in the levels of inflammatory parameters depending on the etiology of erythroderma. The median level of erythrocyte sedimentation rate (ESR) was highest in patients with erythrodermic psoriasis, and ESR levels were significantly higher compared to patients with erythroderma caused by atopic dermatitis. C-reactive protein (CRP), on the other hand, was significantly higher in the subjects with drug-induced erythroderma than in those with primary cutaneous lymphomas. The median CRP level was highest in patients with drug-induced erythroderma. Ohga et al. described high CRP levels in patients with psoriatic erythroderma and generalized drug eruption [13]. On the other hand, Auastad et al. and César et al. observed no correlation between etiology and CRP or ESR in their studies [3,5].

The median LDH level was the highest in patients with AD erythroderma; however, no statistical significance was observed. The available literature does not provide clear reports on this topic. Miyashiro and Sanches showed widespread elevation of LDH in patients with erythrodermic CTCL [2]. This is an important finding because, as is well known, elevated LDH levels are considered a poor prognostic factor. In contrast, the elevation of LDH has been reported far less frequently in patients with psoriasis. Yet, not every retrospective study on erythroderma includes an analysis of those inflammatory parameters. More research on this topic is needed.

As mentioned above, we showed that IgE levels were significantly higher in patients with AD compared to other etiologies. We have discussed this issue in more detail earlier in this article. The second-highest median IgE levels occurred in patients with CTCL erythroderma. In this particular group, IgE levels were tested in 40.74% of the subjects. Interestingly, among those tested, as many as 72.73% showed IgE levels high enough to be second only to the aforementioned AD. Nevertheless, there was no statistical significance in this case. There are few reports in the literature on IgE levels in patients with CTCL erythroderma. Miyashiro and Sanches reported that total IgE levels were above the normal range in all CTCL patients, but values were not pronounced enough to be a significant finding, while Khaled et al. found that significantly increased IgE levels were present in 25% of patients with CTCL-induced erythroderma [2,31].

We found no statistically significant difference between the levels of other laboratory parameters in peripheral blood smear according to etiology. Yet, some correlations can be found in the literature. Miyashiro and Sanches found that the median leukocyte count and median lymphocyte count were higher in SS [2]. The median eosinophil count was higher in AD patients and idiopathic causes of erythroderma. César et al. showed that eosinophilia is correlated with malignancy-induced erythroderma [5].

### 4.10. Comorbidities

We analyzed the prevalence of comorbidities in our study group. The most common were hypertension, cardiovascular diseases, hematological diseases, and diabetes. There is limited information on comorbidities in patients with erythroderma in the available literature. In a study of 47 patients with erythroderma, 14 had hypertension, 12 had diabetes, and four suffered from heart disease [8]. Ma et al. found that metabolic syndrome and component conditions (hypertension, abdominal obesity, dyslipidemia) are more frequent in patients with erythrodermic psoriasis [41]. The authors reported that metabolic syndrome is an independent predictor of erythrodermic psoriasis and may increase the risk of cardiovascular disease, which is relatively common in this condition. A few cases of erythroderma in patients with hematological diseases can also be found in the literature [42,43]. Moreover, it can lead to serious complications such as heart failure, in which case elderly patients with heart disease are of special concern [7,44].

### 4.11. Erythroderma in Children

In general, erythroderma in children is a rare symptom. Our results reflect the global trend, indicating that erythroderma in children occurs much less frequently than in adults. However, it is important to note that, as mentioned earlier, the sample size was inadequate for statistical analysis. Aside from that, children are hospitalized in our dermatology department from 2 years old, so this study does not include data on newborns and infants that constitute a large percentage of the youngest patients with erythroderma. In the pediatric population of our study, the leading cause of erythroderma was AD (62.5%). Although AD is mentioned as one of the causative agents in the available literature, the authors do not agree on the main underlying factor [45,46,47,48]. Among other causes of erythroderma in children, our study identified individual cases of psoriatic, idiopathic, and drug-induced erythroderma. These causes have also been reported in the literature [45,46,47,48,49]. The drug reaction in our study was attributed to the administration of terbinafine, prescribed for a fungal lesion on the buccal mucosa. Erythroderma in children also occurs as a result of exacerbation of genodermatoses; no cases of this nature were identified in our study [45,49]. The treatment included primarily systemic and topical steroids and antihistamines. Phototherapy was used for erythroderma associated with AD and antibiotics in a case of idiopathic erythroderma. This topic deserves more attention if only because the erythrodermic course of skin diseases in children can be life-threatening. More recent studies on larger populations are needed.

### 4.12. Treatment

The management of erythroderma in our patients varied depending on the underlying cause and was in accordance with the literature [1,3,7]. Topical steroids and systemic antihistamines were used first in most patients on admission. During the hospitalization, treatment was determined by the primary cause and included topical and systemic steroids, systemic antihistamines, methotrexate, acitretin, and cyclosporine A. Patients with elevated CRP and neutrophil levels were significantly more likely to receive antibiotics. The most common systemic treatments were corticosteroids and systemic antihistamines. The only treatment we used that seems to be controversial is the recommendation of systemic steroids for patients with erythrodermic psoriasis. Some consider systemic steroids as a factor that may lead to exacerbation of psoriasis and even trigger an erythrodermic reaction [3,11]. On the other hand, in current scientific publications, one can also find papers that report the use of oral steroids in erythrodermic patients diagnosed with plaque psoriasis for a short time [8]. Of our 49 patients with erythroderma due to psoriasis, 17 also received short-term systemic steroids as part of their treatment, and what is more important—with a satisfactory therapeutic effect. However, we want to emphasize that systemic steroids were used in a significant minority of patients with erythrodermic psoriasis and less frequently compared to patients with other erythroderma causes. Lo Y. and Tsai T.-F. report that the use of systemic steroids in the exacerbation of psoriasis vulgaris may improve the disease and that this treatment is more common worldwide than it seems [12]. It makes the topic still very controversial. Therefore, further studies in this direction with more people tested are needed.

An interesting group was the group of patients with idiopathic erythroderma, in which, in addition to systemic antihistamines, systemic steroids, antibiotics, as well as immunosuppressive drugs were most often used. This treatment was also consistent with the literature [1,50].

Biologic therapy is a promising therapeutic option for some skin diseases, including psoriasis and atopic dermatitis. The literature provides information on the treatment of erythroderma with biological drugs. Potestio et al. conducted a meta-analysis on biologic therapy in erythrodermic psoriasis [51]. The authors concluded that despite limited data, this type of treatment appears to be effective, emphasizing treatment with IL-17 and IL-23 [51]. This is supported by Avallone et al., who conducted a multicenter, retrospective study to compare the treatment of pustular and erythrodermic psoriasis with IL-17 and IL-23 inhibitors [52]. The authors assumed that both drugs were effective in treating these dermatoses [52]. There are fewer reports on the treatment of erythrodermic atopic dermatitis, but some studies have shown the efficacy of dupilumab in treating erythrodermic atopic dermatitis [53,54].

### 4.13. Limitations

We are aware of the limitations of our study. First of all, as we conducted a retrospective study, we only depended on patients’ medical records, and we did not have an opportunity to examine the patients. We faced incomplete information in some of the patients’ records as well. While we have identified several cases of erythroderma in children, the sample size remains insufficient for drawing definitive conclusions. Nevertheless, we believe that this study provides important findings that support previous reports in the literature.

## 5. Conclusions

Erythroderma is a serious symptom and a potentially life-threatening condition affecting patients at any age. It can be caused by several factors, but it is most often caused by an exacerbation of preexisting skin disorder, predominantly plaque psoriasis, atopic dermatitis, and cutaneous T-cell lymphomas. A particular challenge is the diagnosis and treatment of idiopathic erythroderma, which requires extensive tests, including histopathological examinations and laboratory results. The condition of erythroderma usually requires prolonged hospitalization and intensive treatment, which is also a big challenge for health insurance systems.

## Figures and Tables

**Table 1:** Characteristics of patients according to the most common etiology of erythroderma.

Etiology			Plaque Psoriasis	AD	CTCL	Drug Reaction	Eczema	Idiopathic
			*n* = 49 (24.02%)	*n* = 27 (13.24%)	*n* = 27 (13.24%)	*n* = 22 (10.78%)	*n* = 21 (10.29%)	*n* = 39 (19.12%)
Median age (Q1–Q3)			51 (41–61)	40 (21–54)	66 (59–77)	67 (58–72)	68 (63–73)	64 (59–70)
Hospital stay in days (median)			10	9	9	10	13	10
Laboratory parameters	Erythrocytes	Number of patients	48/49	27/27	27/27	22/22	21/21	37/39
		Median cells/mm^3^ (Q1–Q3)	4.29 (3.9–4.49)	4.58 (4.3–4.85)	4.37 (3.68–4.64)	4.1 (3.7–4.53)	3.98 (3.72–4.33)	4.06 (3.47–4.4)
	Leukocytes	Number of patients	48/49	27/27	27/27	22/22	21/21	37/39
		Median cells/mm^3^ (Q1–Q3)	9.35 (7.91–11.76)	8.88 (7.61–10.56)	9.01 (6.55–10.82)	10.195 (8.55–14.84)	8.87(8.35–10.61)	9.11(7.65–13.73)
	Lymphocytes	Number of patients	47/49	27/27	26/27	22/22	21/21	35/39
		Median cells/mm^3^ (Q1–Q3)	1.79 (1.35–2.58)	1.82 (1.26–2.88)	1.86 (1.35–4.43)	2.23 (1.23–2.61)	1.69 (1.09–2.14)	1.88 (1.43–2.42)
	Neutrophils	Number of patients	47/49	27/27	26/27	22/22	21/21	35/39
		Median cells/mm^3^ (Q1–Q3)	5.93 (4.67–8.54)	5.84 (4.81–6.93)	4.43 (3.01–5.98)	5.87 (4.35–9.29)	6.35 (4.67–7.67)	5.7 (4.13–9.07)
	Eosinophils	Number of patients	47/49	27/27	25/27	22/22	21/21	35/39
		Median cells/mm^3^ (Q1–Q3)	0.26 (0.06–0.48)	0.28 (0.06–0.86)	0.23 (0.07–0.52)	0.33 (0.05–1.58)	0.15 (0.02–0.24)	0.3 (0.07–0.99)
	ESR	Number of patients	40/49	18/27	24/27	17/22	16/21	35/39
		Median mm (Q1–Q3)	28.5 (12–47)	10.5 (7–17)	17 (8–26)	12 (8–16)	19 (7.5–27)	25 (14–35)
	CRP	Number of patients	47/49	27/27	26/27	22/22	21/21	37/39
		Median mg/L (Q1–Q3)	28.6 (7.5–48.5)	8.4 (2.7–20)	6.05 (1.6–11.6)	28.7 (5.7–88.9)	11.4 (4.7–42)	21 (5–34)
	Procalcitonin	Number of patients	11/49	2/27	1/27	8/22	1/21	5/39
		Median μg/L (Q1–Q3)	0.06 (0.03–0.08)	0.05 (0.04–0.05)	0.03	0.115 (0.05–0.31)	0.15 (0.15–0.15)	0.08 (0.01–0.09)
	IgE total	Number of patients	6/49	23/27	11/27	10/22	14/21	21/39
		Median U/mL (Q1–Q3)	90.6 (32.9–542)	19,100 (6430–32,300)	1870 (183–4240)	45.1 (11–111)	200 (147–1090)	943 (330–2160)
	Albumin	Number of patients	29/49	14/27	20/27	17/22	13//21	21/39
		Median mg/mL (Q1–Q3)	3.7 (3.5–3.9)	4 (3.6–4.3)	3.85 (3.5–4)	3.3 (3.1–3.9)	3.6 (3.3–3.9)	3.4 (2.8–4)
	LDH	Number of patients	14/49	12/27	21/27	15/22	16/21	21/39
		Median U/L (Q1–Q3)	202.5 (174–222)	303.5 (242–372)	252 (222–279)	254 (199–413)	245 (204.5–313.5)	291 (226–376)
	β2-Microglobulin	Number of patients	3/49	6/27	23/27	8/22	11/21	15/39
		Median mg/L (Q1–Q3)	2.67 (2.16–2.68)	2.38 (1.9–2.7)	3.2 (2.57–3.84)	5.555 (3.04–8.73)	3.32 (2.28–4.07)	3.06 (2.49–5.52)
Comorbidities	Hypertension		18 (36.73%)	5 (18.52%)	11 (40.74%)	11 (50%)	7 (33.33%)	20 (51.28%)
	Heart diseases		9 (18.37%)	4 (14.81%)	7 (25.935)	6 (27.27%)	4 (19.05%)	8 (20.51%)
	Hematological diseases		5 (10.20%)	2 (7.41%)	4 (14.81%)	5 (22.73%)	5 (23.81%)	12 (30.77%)
	Diabetes		5 (10.20%)	2 (7.41%)	5 (18.52%)	6 (27.27%)	4 (19.05%)	10 (25.64%)

*n*—number of patients; ESR—erythrocyte sedimentation rate; CRP—C-reactive proteine; IgE—immunoglobulin E; LDH—Lactate dehydrogenase; AD—atopic dermatitis; CTCL—primary cutaneous T-cell lymphoma.

## Data Availability

Data are contained within the article and Supplementary Materials.

## Supplementary Materials

The following supporting information can be downloaded at: https://www.mdpi.com/article/10.3390/jcm13030645/s1, Table S1: Characteristics of patients with erythroderma according to rare etiologies; Table S2: Detailed characteristics of drug-induced erythroderma patients.
