# Supplementary material for: Erythroderma: A Retrospective Study of 212 Patients Hospitalized in a Tertiary Center in Lower Silesia, Poland
Source: J Clin Med. 2024 Jan 23;13(3):645. doi: 10.3390/jcm13030645 (PMC10856417; doi:10.3390/jcm13030645)
Supplement: Supplementary file 1 [file jcm-13-00645-s001.zip › jcm-2804107-supplementary.pdf]

Table S1. Characteristics of patients according to rare etiologies of erythroderma.

Etiology			PRP	GPP	TEN/SJS	Congenital ichthyosis	PF	Crusted scabies	Seborrheic dermatitis
			n = 8 (3.92%)	n = 4 (1.96%)	n = 2 (0.98%)	n = 2 (0.98%)	n = 1 (0.49%)	n = 1 (0.49%)	n = 1 (0.49%)
Median age (Q1-Q3)			57.5 (51-71)	64 (59.5-68.5)	68 (59-77)	64 (48-82)	56 (56-56)	27(27-27)	74 (74-74)
Hospital stay in days (median)			15	12.5	14.5	12.5	24	4	7
Laboratory parameters	Erythrocytes	Number of patients	8/8	4/4	2/2	2/2	1/1	1/1	1/1
		Median cells/mm^3 (Q1-Q3)	4.67 (3.97-5.02)	4.63 (4.05-4.85)	4.2 (3.5-4.689)	4.1 (3.29-4.9)	4.89 (4.89-4.89)	4.25 (4.25-4.25)	3.86 (3.86-3.86)
	Leukocytes	Number of patients	8/8	4/4	2/2	2/2	1/1	1/1	1/1
		Median cells/mm^3 (Q1-Q3)	10.97 (9.37-12.54)	16.99 (13.09-21.84)	12.47 (1.88-23.06)	12.32 (6.64-17.99)	14.45 (14.45-14.45)	10.62 (10.62-10.62)	8.18 (8.18-8.18)

Lymphocytes	Number of patients	7/8	4/4	2/2	1/2	1/1	1/1	1/1
	Median cells/mm^3 (Q1-Q3)	2.39 (2.24-3.47)	2.21 (1.62-3.07)	0.52 (0.43-0.6)	1.3 (1.3-1.3)	3.3 (3.33-3.33)	2.41 (2.41-2.41)	2.72 (2.72-2.72)
Neutrophils	Number of patients	7/8	4/4	2/2	1/2	1/1	1/1	1/1
	Median cells/mm^3 (Q1-Q3)	6.31 (5.81-7.7)	13.77 (9.27-17.905)	10.955 (1.22-20.77)	15.89 (15.89-15.89)	9.76 (9.76-9.76)	5.84 (5.84-5.84)	4.67 (4.67-4.67)
Eosinophils	Number of patients	7/8	4/4	2/2	1/2	1/1	1/1	1/1
	Median cells/mm^3 (Q1-Q3)	0.65 (0.1-1.49)	0.2 (0.115-0.66)	0(0-0)	0.3 (0.3-0.3)	0.27 (0.27-0.27)	1.66 (1.66-1.66)	0.11 (0.1-0.11)
ESR	Number of patients	5/8	4/4	1/2	1/2	1/1	1/1	1/1

	Median mm (Q1-Q3)	22 (20-49)	28.5 (26.5-88)	21 (21-21)	59 (59-59)	61 (61-61)	33 (33-33)	17 (17-17)
CRP	Number of patients	7/8	4/4	2/2	2/2	1/1	1/1	1/1
	Median mg/l (Q1-Q3)	3.4 (1.5-5.6)	116.65 (89.6-208.65)	99.3 (79.3.6-119.3)	33.1 (0.3-65.9)	58.5 (58.5-58.5)	31.8 (31.8-31.8)	1.3 (1.3-1.3)
Procalcitonin	Number of patients	1/8	4/4	2/2	1/2	0/1	0/1	0/1
	Median µg/l (Q1-Q3)	0.63 (0.63-0.63)	0.11 (0.055-0.72)	1.04 (0.29-1.79)	0.24 (0.24-0.24)	-	-	-
IgE total	Number of patients	2/8	0/4	0/2	0/2	0/1	0/1	0/1
	Median U/mL (Q1-Q3)	6829.6 (958-12700)	-	-	-	1850 (1850-1850)	-	-
Albumin	Number of patients	6/8	3/4	2/2	1/2	0/1	0/1	1/1
	Median mg/ml	3.85 (3.7-4.2)	3.2 (3.2-3.7)	2.95 (2.8-3.1)	2.5 (2.5-2.5)	-	-	3.6 (3.6-3.6)

	(Q1-Q3)							
LDH	Number of patients	3/8	2/4	1/2	1/2	0/1	0/1	1/1
	Median U/l (Q1-Q3)	191 (162-307)	224 (207-241)	751 (751-751)	378 (378-378)	-	-	176 (176-176)
β2-Microglobulin	Number of patients	2/8	0/4	0/2	1/2	1/1	0/1	0/1
	Median mg/l (Q1-Q3)	5.195 (2.43-7.96)	-	-	3.75 (3.75-3.75)	3.13 (3.13-3.13)	-	-

n – number of patients; ESR – erythrocyte sedimentation rate; CRP – C-reactive protein; IgE – immunoglobulin E; LDH - Lactate dehydrogenase; PRP – pityriasis rubra pilaris; GPP – generalized pustular psoriasis; TEN/SJS – toxic epidermal necrolysis/ Stevens-Johnson syndrome; PF - pemphigus foliaceus

Table S.2. Detailed characteristics of drug-induced erythroderma patients.

Patient	Age and gender	Drug suspected for erythroderma induction	Timing of the erythroderma development post-drug administration	Other drugs administered before admission
1.	59 F	Lamotrigine	ND	Omeprazole<br>Venlafaxine<br>Levomepromazine
2.	54 M	Venlafaxine	After around 2 weeks post-drug administration	Metformin<br>Simvastatin<br>Carbamazepine<br>trazodone
3.	72 M	Carbamazepine	5 days post-drug administration	Nebivolol<br>Atorvastatin<br>Amlodipine<br>aspart insulin
4.	60 M	Fenofibrate	ND	Omeprazole<br>Tamsulosin<br>Atorvastatin
5.	52 M	Telaprevir	After approximately 3 months of treatment	Peg-Interferon α-2a<br>Ribavirin
6.	58 M	Carbamazepine	Approximately 7 days post-drug administration	
7.	25 F	Carbamazepine	Less than 7 days post-drug administration	-
8.	81 F	Allopurinol	ND	Acetylsalicylic acid<br>Paroxetine<br>Torsemide<br>Propylthiouracil
9.	84 F	Allopurinol	7 days post-drug administration	Amlodipine<br>Ramipril
10.	83 F	Terbinafine	5 days post-drug administration	-
11.	67 F	Allopurinol	7 days post-drug administration	Bortezomib, thalidomide, dexamethasone – 2nd dose administered 11 days before erythroderma

12.	67 F	Allopurinol	3 days post-drug administration	Metformin<br>Telmisartan<br>Indapamide<br>Chondroitin sulfate
13.	67 F	Allopurinol	1-2 weeks post allopurinol administration	Ibandronic acid<br>Glargine insulin<br>Nitrendipine<br>Hydroxyzine
14.	79 M	Bromhexine	Less than 7 days post-drug administration	Immunoglobulins<br>Metformin<br>Gliclazide<br>Atorvastatin<br>Acetylsalicylic acid<br>Carbamazepine<br>Duloxetine<br>Vinpocetine
15.	68 M	Tolperisone	7 days post-drug administration	Valsartan<br>Hydrochlorothiazide<br>Pantoprazole<br>Torsemide<br>Allopurinol<br>Nebivolol
16.	*72 M*	*Norfloxacin*	*2 days post-drug administration*	*Dabigatran*<br>*Metformin*<br>*Lizpro insulin*<br>*Valsartan*<br>*Betaxolol*<br>*Torsemide*<br>*Atorvastatin*<br>*Isosorbide*<br>*Trimebutine*
17.	*62 F*	*Tizanidine*<br>*Sulfasalazine*<br>*Pregabalin*	*Drugs were administered for around a month*	-

18.	*70 F*	*Amoxicillin Allopurinol*	*Less than 7 days post amoxicillin administration; Allopurinol administered several months before erythroderma*	*-*
19.	*39 M*	*Amoxicillin and clavulanic acid and doxycycline*	*4 days post-drugs administration*	*ND*
20.	*64 F*	*Cefuroxime Allopurinol*	*Cefuroxime administered less than 7 days before erythroderma, allopurinol administered for 80 days before erythroderma*	*Spironolactone Betahistine Perindopril Bisoprolol Atorvastatin Opipramol Nitrazepam*
21.	*82 F*	*Allopurinol Venlafaxine*	*13 days post venlafaxine administration and 6 days post allopurinol administration*	*Zolpidem Lorazepam Quetiapine Valsartan Chlorthalidone Bisoprolol*
22.	*51 M*	*Acenocoumarin Warfarin*	*ND*	*Rivaroxaban Metformin*

In cases 16-22 highlighted in italics, based on medical documentation, the authors were unable to definitively determine the causative agent of erythroderma. Due to insufficient data regarding the medications taken by patients or the concurrent use of multiple medications, it is suspected that drug interactions may have contributed to the onset of erythroderma.

F- female; M – male; ND - no data
